# A Lightweight Machine Learning Model for High Precision Gastrointestinal Stromal Tumors Identification

**DOI:** 10.3390/bioengineering12040381

**Published:** 2025-04-03

**Authors:** Xin Sun, Xiwen Mo, Jing Shi, Xinran Zhou, Yanqing Niu, Xiao-Dong Zhang, Man Li, Yonghui Li

**Affiliations:** 1 Haihe Hospital, Tianjin University, Tianjin 300350, China; 13302031999@126.com; 2 Tianjin Union Medical Center, Nankai University, Tianjin 300071, China; 3 Tianjin Key Laboratory of Brain Science and Neural Engineering, Academy of Medical Engineering and Translational Medicine, Tianjin University, Tianjin 300072, China; moxiwen1999@163.com (X.M.); jingshi@tju.edu.cn (J.S.); xinranzhou@tju.edu.cn (X.Z.); xiaodongzhang@tju.edu.cn (X.-D.Z.); 4 Department of Clinical Medicine, Tianjin Medical University, Tianjin 300203, China; nyq18631319813@163.com; 5 Department of Physics and Tianjin Key Laboratory of Low Dimensional Materials Physics and Preparing Technology, School of Sciences, Tianjin University, Tianjin 300350, China; 6 Gastroenterology, The Second Hospital of Tianjin Medical University, Tianjin 300211, China

**Keywords:** a lightweight model, gastrointestinal stromal tumors, endoscopic ultrasound image, high accuracy

## Abstract

Gastrointestinal stromal tumors (GISTs), which usually develop with a significant malignant potential, are a serious challenge in stromal health. With Endoscopic ultrasound (EUS), GISTs can appear similar to other tumors. This study introduces a lightweight convolutional neural network model optimized for the classification of GISTs and leiomyomas using EUS images only. Models are constructed based on a dataset that comprises 13277 augmented grayscale images derived from 703 patients, ensuring a balanced representation between GIST and leiomyoma cases. The optimized model architecture includes seven convolutional units followed by fully connected layers. After being trained and evaluated with a 5-fold cross-validation, the optimized model achieves an average validation accuracy of 96.2%. The model achieved a sensitivity, specificity, positive predictive value, and negative predictive value of 97.7%, 94.7%, 94.6%, and 97.7%, respectively, and significantly outperformed endoscopists’ assessments. The study highlights the model’s robustness and consistency. Our results suggest that instead of using developed deep models with fine-tuning, lightweight models with their simpler designs may grasp the essence and drop speckle noise. A lightweight model as a hypothesis with fewer model parameters is preferable to a deeper model with 10 times the model parameters according to Occam’s razor statement.

## 1. Introduction

Gastrointestinal stromal tumors (GISTs) are common mesenchymal neoplasms that originate from the interstitial cells of Cajal and can occur at any site within the gastrointestinal tract [1]. Major occurrences of GISTs are in the stomach (approximately 60% to 70%) and the small intestine (approximately 20% to 25%) [2]. Since the primary spreading pathways of GISTs are the hematogenous routes, the development of GISTs is a severe threat to the liver and peritoneum. Metastasis is present in up to 47% of cases at diagnosis [3]. As a critical component of the gold standard, immunohistochemical markers play a crucial role in diagnosing GISTs, with the most commonly used markers being c-kit (CD117), DOG1, and CD34. Although CD34 is positive in 60% to 70% of GISTs, its diagnostic utility is limited by its lack of specificity [4,5]. In contrast, c-kit is more sensitive and specific, identifying approximately 95% of GIST cases. The DOG1 antibody has shown a sensitivity ranging from 87% to 97% in differentiating GISTs in various studies [6]. Besides, endoscopic ultrasound-guided fine-needle aspiration (EUS-FNA) or fine-needle biopsy (EUS-FNB) are highly effective diagnostic methods with an accuracy rate of up to 86–95.6% [7,8,9].

But before the time-consuming and tissue-sampling-required diagnosis, endoscopic ultrasound (EUS) image-based diagnosis can usually serve as a low-cost screening tool. EUS images provide subepithelial lesions (SELs) information in the gastrointestinal tract with detailed assessments of lesion size, margins, origin layer, and echogenic characteristics [10].

However, distinguishing GISTs from other SELs, particularly leiomyomas, remains challenging for endoscopists due to their similar EUS imaging features. Leiomyomas typically appear as hypoechoic, homogeneous, well-defined lesions originating from the second or fourth layer of the gastric wall on EUS. GISTs, on the other hand, generally present as hypoechoic, relatively homogeneous masses, also arising from the second or fourth layer of the gastric wall [11]. Several EUS features, such as size, irregular margins, intratumoral cystic spaces, presence of echogenic foci, and peritumoral lymphadenopathy, are considered predictive indicators of malignancy. The diagnostic accuracy of EUS for GISTs has been reported as 77.1% [12], with no significant correlation observed between diagnostic accuracy and the tumor’s location, size, or originating layer. The overall accuracy of EUS imaging for SELs based on gastric lesions is 66.7%, indicating potential limitations in relying solely on imaging characteristics for diagnosis [12].

Due to the significant limitations and inaccuracies inherent in imaging-based diagnostics, there has been an increasing amount of research focused on leveraging artificial intelligence (AI) to enhance diagnostic accuracy and precision [13,14,15,16]. A meta-analysis of seven studies (2431 patients, 36,186 images) reported an AUC of 0.950 for AI-assisted EUS in GIST detection [15], while another study showed an AUC of 0.94 in distinguishing GISTs from other SELs [17]. These findings underscore the high diagnostic capability of AI-based EUS in distinguishing GISTs from other SELs. Recent pilot studies have also assessed AI efficacy in clinical settings, demonstrating that AI models outperform human experts in diagnosing SELs ≥20 mm, with an AUC of 0.965 vs. 0.684 for expert endoscopists (*p* = 0.007) [16]. These results highlight AI’s potential in reducing reliance on invasive biopsy procedures. To improve generalizability, the TN-USMA Net model was developed, incorporating ultrasound-specific pretraining and meta-attention mechanisms, achieving an AUC of 0.881, surpassing conventional approaches [14]. Further advancing AI applications in ultrasound imaging, a deep learning model was developed for the automatic segmentation and risk stratification of GISTs using transabdominal ultrasound images, achieving an AUC of 92.5% for risk prediction while significantly improving lesion delineation accuracy [18]. Beyond EUS, AI has also been applied to histopathological image analysis for stromal tumor diagnosis. The STT-BOX system, trained on H&E-stained pathology images, achieved 100% AUC in GIST classification and demonstrated strong generalizability [19]. In addition to diagnosis, AI models have been used to predict patient prognosis and genetic mutations in GISTs [20]. Multi-modal AI approaches integrating histopathology, radiomics, and clinical data further enhance prognostic predictions, with a recent model achieving a C-index of 0.864 for recurrence-free survival (RFS) in an external validation cohort [21]. These findings underscore the complementary potential of histology-based and EUS-based AI models, emphasizing the need for domain-specific architectures.

Previous works display great success in the AI-based EUS reading, but there seems to be a divergence between general deep learning image recognition algorithms and the patterns in EUS images. First, it is difficult to believe that patterns in GIST/leiomyomas are covered in general models (ResNet, Efficientnet, etc.). Second, compared to the multipurpose image identification tasks, EUS images are bound to equipment-determined shapes. So, it may not be necessary to maintain the same structure as the general models. Last, limited by the EUS image dataset, designing a task-focused lightweight model may be the best approach. This study aims to design, optimize, and explore the boundary of lightweight models in AI-assisted EUS diagnosis of GISTs. According to Occam’s Razor, lightweight models may be the best practical approach in EUS diagnosis for their complementary components.

## 2. Materials and Method

### 2.1. Description and Details of the Dataset

The dataset comprises EUS images collected from 681 patients at The Second Hospital of Tianjin Medical University between June 2014 and October 2020. As the most difficult of EUS images in the classification of GIST and leiomyoma images, there are 403 GIST images from 253 patients and 420 leiomyoma images from 428 patients in the dataset after data cleaning. Besides, to test the lightweight model, an independent test set is compiled with the latest cases from the same hospital between 2022 and 2024, comprising 66 images of GISTs from 11 patients and 43 images of leiomyomas from 11 patients. The test set is completely independent of the training and validation sets to avoid information leakage. Multiple images from the same patient are considered for taking EUS images with variability in lesion appearance, orientation, and other characteristics. There is no mathematical correlation between images from the same patients. Based on the correlation of each image pair, there is no clear clue to tell images from the same patient apart from the whole dataset.

To guarantee the quality of the dataset, images and labels are double-checked to drop problematic ones. Bad images include ones with low resolution, excessive noise, or artifacts. Bad labels include ambiguous pathological diagnoses. For instance, ambiguous diagnoses can be “gene tests suggest to rule out GIST before considering leiomyoma” or “gene testing depends on the exclusion of GIST”. Unfortunately, based on current medical conditions, there is no accurate way to validate them.

Unclear images or ambiguous diagnoses are excluded before model training. Unclear images are mainly due to patients’ movement, EUS artifacts, and poor mechanical condition of the EUS sensor with excessive noise. Since the GIST/non-GIST labels are determined by a set of 7 biomarkers, several results with contradicted results. The labels are ensured through pathological examination following surgical or endoscopic resection.

**Figure 1:**
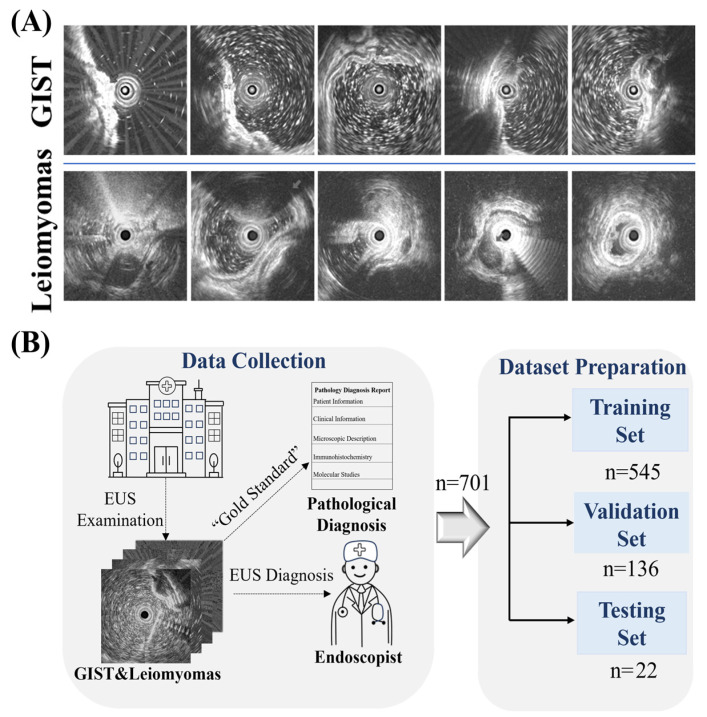
Overview of dataset images and study workflow. (**A**) typical images in the constructed dataset: typical EUS images of GISTs (the top row) and leiomyomas (the bottom row) showing different images after data augmentation. (**B**) Dataset construction including hospital data collection, and diagnosis report with endoscopist review. The data are randomly split into training and validation sets, with an additional independent test set collected separately for model evaluation.

Table 1 summarizes the demographic and clinical characteristics of patients in the training, validation, and test datasets. The average age is 56.26 ± 9.89 years for the training and validation dataset and 57.59 ± 10.74 years for the test dataset. Females account for 63.4% and 54.5% of patients in the respective datasets. The average lesion size is 9.3 ± 5.7 mm in the training and validation dataset, with 93.4% of lesions ˂20 mm. In the test dataset, the average lesion size is 18.7 ± 10.7 mm, with 63.64% of lesions ˂20 mm. Lesions are primarily located in the gastric fundus (49.5%) and body (12.5%) in the training and validation dataset, while the test dataset shows similar trends with 50% in the fundus and 27.3% in the body. Most lesions are found in the muscularis propria (84% in the training and validation dataset, 86.4% in the test dataset), with fewer in the mucosa and submucosa. Typical images are selected and displayed in Figure 1A.

Besides, as displayed in Figure 1B, the preparation and constitution of the dataset span a period of 8 years (2014–2020, 2022–2024). A training set and a validation set, in a 7:3 ratio, are split from the dataset of 681 patients. As we progressively developed the model, data from 22 patients were collected independently in the period of 2022–2024 as the test set. The collection and research with the dataset are approved by the Medical Ethics Committee of The Second Hospital of Tianjin Medical University under permit number KY2020K140. In practice, since endoscopists have difficulty diagnosing large lesions, the composed test dataset coincidentally includes more large lesion cases (see lesion size statistics). Thus, the proposed lightweight model is challenged for its potential in clinical services.

### 2.2. Data Augmentation and Evaluation Metrics

Due to the rotational symmetry of EUS images, rotational data augmentation is adopted in this study to support model training. Concretely, the rotation is applied before trimming to avoid blank corners in each augmented image to prevent feature extractors from capturing unnecessary features. The resolution of each raw image is at least 500 × 400. The raw image is then rotated by 22.5-degree increments around the center of the ultrasound probe, which expands each image into 15 more variations. All images are then trimmed into 360 × 360 resolution without scaling. Since the 360 × 360 trimming box is smaller than the raw image in all directions, the blank corners can be avoided. After augmentation, the expanded dataset consisted of 6448 GIST images and 6720 leiomyoma grayscale images. Then, by flattening all images into a one-dimensional array, the mean and standard deviation of the whole dataset are found to be 0.4093 and 0.2018 for the entire set, respectively.

The model is trained using backpropagation and model parameter optimization employing the Adam optimizer with an initial learning rate of 5 × 10^−5^ and a batch size of 32. To boost the convergence, a scheduler is used to reduce the learning rate by half every 250 epochs during training. The cross-entropy loss is incorporated. The model and algorithm are developed and implemented in the Python 3.9.17 environment using the PyTorch 2.0.1 framework, running on Windows/Linux platforms. All computations are performed on a high-performance computing server equipped with dual NVIDIA GeForce RTX 3090 GPUs (USA).

To comprehensively evaluate the performance of the convolutional neural network (CNN) in the classification task, various performance metrics are used, including accuracy, sensitivity, specificity, positive predictive value (PPV), negative predictive value (NPV), F1 score, and the area under the receiver operating characteristic (ROC) curve (AUC). Accuracy and sensitivity are measured by calculating the proportion of correctly predicted samples.

## 3. Results

### 3.1. Performance of the Lightweight Model

**Figure 2:**
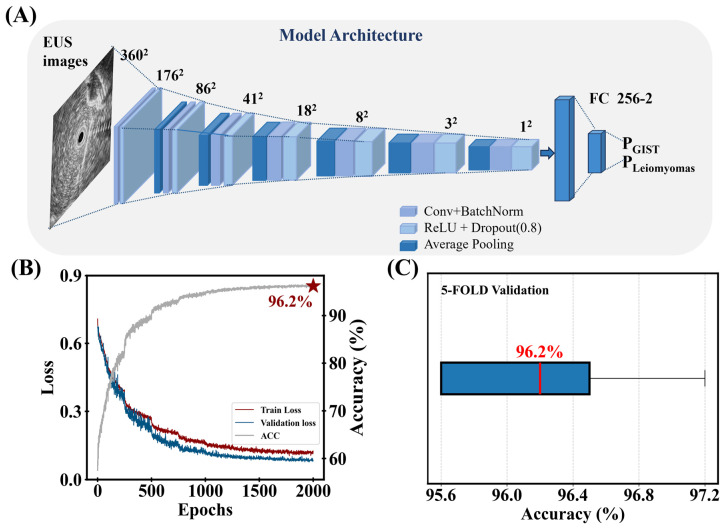
(**A**) The model architecture with its network details is shown. There are seven convolutional units in the network. Each convolutional unit contains a convolutional layer, a batch normal layer, and an average pooling layer. After the seven convolutional units, extracted features are input into several fully connected layers for final classification. (**B**) The average training loss, validation loss, and accuracy curves from 5-fold cross-validation. Asterisk represents the accuracy value that the model can reach on the validation set. (**C**) A box plot for the performance statistics of the 5-fold cross-validation.

The best lightweight model is presented in Figure 2A with its validated architecture displayed. A detailed summary of the model’s layer-wise parameters, including kernel sizes, strides, and channel counts, is provided in Table 2.

As we shall emphasize later, the dropout layers are important ingredients in the EUS recognition tasks. Based on the tuned architecture, a 5-fold cross-validation procedure is applied to avoid bias in the model and the model with a median performance is then selected as the most reliable model (named as “the lightweight model” in the following context including further evaluation). As shown in Figure 2B, the loss and accuracy curves are obtained by averaging the results of 5-fold cross-validation. Over 2000 epochs, the final average accuracy reached 96.2%. The steep drop in the loss curve and a significant rise in the accuracy curve (Figure 2B) demonstrate the success of the scheduler in converging the model at the 2000th epoch. The accuracy distribution across the five folds is shown in Figure 2C, with individual accuracies of 97.2%, 96.5%, 96.2%, 95.6%, and 95.6%. As mentioned above, the lightweight model is the trained model with 96.2% accuracy.

As quantified by various metrics, the lightweight model shows good performance. According to Table 3, by the validation set, the model’s sensitivity, specificity, PPV, and NPV for distinguishing GISTs from non-GISTs are 97.7%, 94.7%, 94.6%, and 97.7%, respectively. In addition, by the test set, on a per-image basis, the model achieved a sensitivity of 93.9%, specificity of 95.4%, accuracy of 94.5%, positive predictive value (PPV) of 96.9%, and negative predictive value (NPV) of 91.1%. Though the performance of the lightweight model on the test set is worse than its performance on the validation set, we are not surprised as the lesion size of the test set is significantly larger. The results also show the robustness and the application potential of the lightweight model. In comparison, the endoscopists’ sensitivity, specificity, PPV, and NPV for distinguishing GISTs from non-GISTs are 55.6%,79.6%, 73.2%, and 64.2%, respectively. These results further highlight the lightweight model’s superior ability to distinguish between GISTs and non-GISTs. The performance of the lightweight model surpasses the performance of human endoscopists.

**Figure 3:**
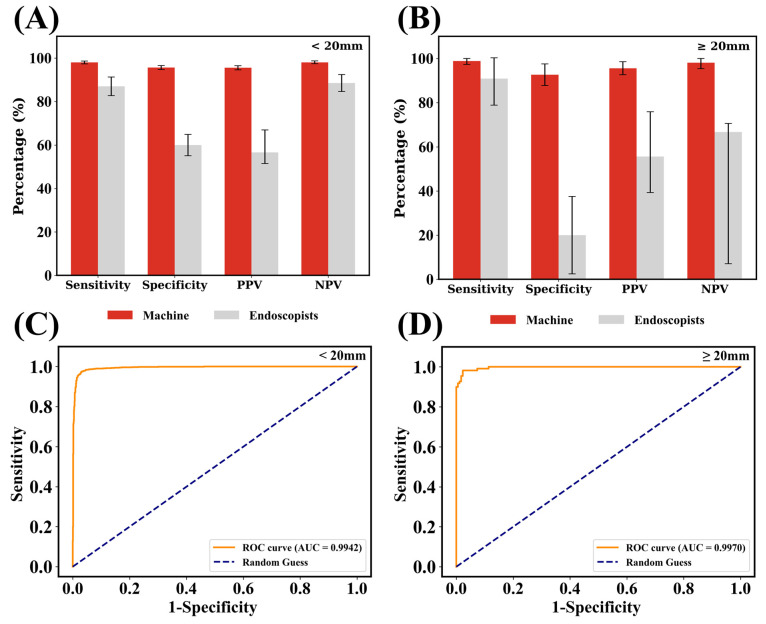
The performance of the best lightweight model by tumor size. (**A**) Forest plot of NPV, PPV, specificity, and sensitivity for endoscopists and machine discrimination of lesions ≤ 20 mm. (**B**) Results for lesions ≥ 20 mm. ROC-AUC for the model grouped by tumors (**C**) smaller than 20 mm and (**D**) larger than 20 mm. Compared to a human’s confidence interval, the confidence interval of the machine is too small to show.

To further compare performance differences between machines and humans, statistics are broken down by tumor sizes. As shown in Figure 3A, the performance of the algorithm behaves uniformly with small standard deviations regardless of the tumor sizes in all statistical indicators. However, the performance of humans depends on the tumor size. When the tumor is smaller than 20 mm, human judgment exhibited a sensitivity of 0.87, specificity of 0.6, PPV of 0.566, and NPV of 0.885. The high NPV value, together with the low PPV value, indicates that the endoscopist usually reports the ambiguous cases as positive ones to not miss any GIST case. Such endoscopists’ behavior can also be represented by the combination of the high sensitivity and the low specificity. In contrast, when the tumor is large (≥20 mm), endoscopists usually face difficulties; it is hard to believe a large tumor to be harmless. Thus, one can see the sensitivity of 0.909, the specificity of 0.2, the PPV of 0.556, and the NPV of 0.667. In addition, Figure 3B,C presents the ROC curves and corresponding AUC values for machine judgment across different tumor size categories. The ROC-AUC for small and large tumors are 0.9942 and 0.997, respectively. The lightweight model shows its potential to consistently make good predictions.

### 3.2. Visualization of the Lightweight Model

**Figure 4:**
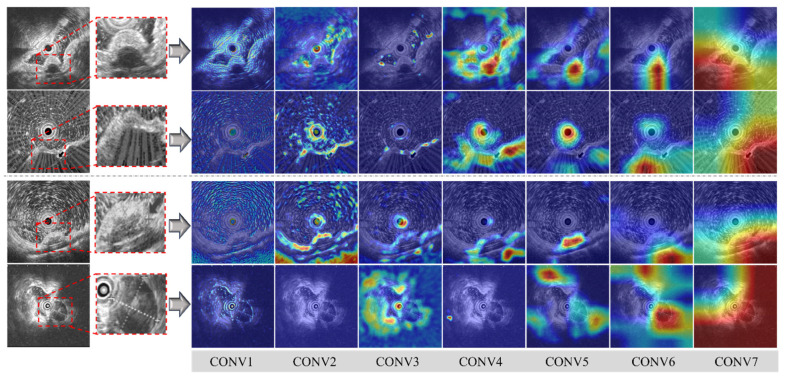
Grad-CAM visualizations of false positive and false negative cases as identified by endoscopists. Row 1 and 2: two false positive cases. Row 3 and 4: two false negative cases.

As the lightweight model outperforms endoscopists, the Grad-CAM is applied to visualize the reason behind the model’s better decisions. As shown in Figure 4, rows 1–2 show two false positive samples, where leiomyomas are misclassified as GISTs by the endoscopists while rows 3–4 display two false negative samples, where GISTs are incorrectly identified as leiomyomas. The model grasps all the details rather than focusing on a small portion of each image with information gathered and compiled in the later convolutional layers. For example, the first false negative case (row 3) is particularly challenging, as it depicts a tumor located in the fundus, which is confirmed through immunohistochemistry as a GIST with leiomyomatous differentiation. In the first two feature extraction layers (CONV1–2) of the model, high-echogenicity regions (enhanced contrast areas in EUS) are traced with a significant number of details. In the intermediate layers (CONV3–5), the features are further compacted by the model, enabling tumor boundaries and the circular probe center to be focused. By the final convolutional layer (CONV6), the core of the lesion is eventually discovered.

In contrast, our endoscopists primarily relied on three criteria: (1) Tumor location, with tumors found in the esophagus or near the cardia generally identified as leiomyomas, while those located in the stomach are typically considered to be GISTs; (2) Tumor size, with larger tumors more likely to be classified as GISTs; and (3) Tumor echo homogeneity, as heterogeneous echoes are often associated with malignant tumors, which increases suspicion of GISTs. On the precision of EUS image reading, the lightweight model can harvest more details than our endoscopist. As indicated by the visualizations, the model extracts more features than humans, which supports better decisions with abundant and non-biased details. Our endoscopists’ experience may sometimes become biased in EUS reading with limited time and resources.

## 4. Discussion

### 4.1. Explorations in Model Architectures

The dataset allows free exploration of lightweight model architectures where the number of layers, convolutional layers, activation functions, overfitting preventing approaches, and other options can be customized. Specifically, since each 2 × 2 pooling layer halves its input in length, the maximum number of convolutional-activation-pooling units can be roughly estimated as $\log_{2}360\approx 8$. Additionally, the number of channels and the kernel size of each convolutional layer are critical parameters to tune in model design.

**Figure 5:**
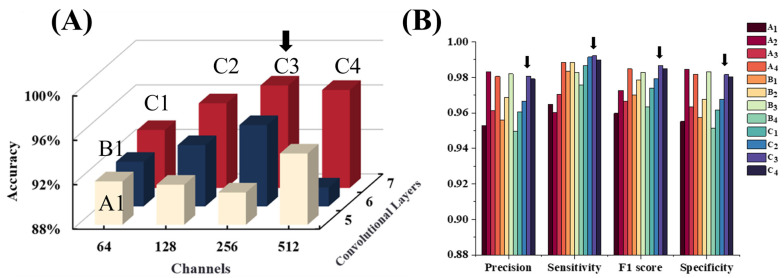
(**A**) The best accuracy achieved during training for the 12 models with different convolutional layer counts (5, 6, 7) and channel numbers (64, 128, 256, 512). Each bar represents the highest accuracy achieved after model convergence under the corresponding settings, with red representing 7-layer networks, blue representing 6-layer networks, and beige representing 5-layer networks. (**B**) Performance of the 12 models across multiple metrics, including precision, sensitivity, F1 score, and specificity.

Extensive evaluation is applied to three primary CNN configurations (A, B, C), each with four variants (A1–A4, B1–B4, C1–C4), resulting in a total of 12 different model architectures. Configuration A, B, and C correspond to 5, 6, and 7 convolutional units respectively. Numbers 1–4 correspond to the number of feature channels of 64, 128, 256, and 512 after all convolutional units, respectively. After 2000 epochs of training, results are summarized in Figure 5A. Additionally, the 512-channel configuration may be overly complex, causing the model to memorize training set details rather than learn generalizable features, thus reducing its performance on the validation set. The lightweight models show their excellent performance and indicate that deep models may not be complementary components for EUS-related tasks.

The detailed metrics are presented in Figure 5B, which indicates the lightweight model could be a competitive model in different aspects. The C3 model achieved a sensitivity greater than 99%, indicating exceptional performance in identifying true positive cases, which is crucial for minimizing the false negative rate of GISTs. From the perspective of precision and specificity, the C3 model also demonstrated high reliability, accurately classifying samples as GISTs while maintaining high specificity values to ensure the accurate identification of leiomyomas, thereby reducing false positives. These results indicate that the C3 model not only efficiently identifies positive samples (GISTs) but also accurately excludes negative samples (leiomyomas) with a low false positive rate, showcasing the best overall performance.

### 4.2. Remark: Which Factors Benefit the Lightweight Model?

Though there are many EUS reading algorithms reported in the literature, a lightweight model may still be a practically useful model with extra insights provided for better practices. To obtain further insights, one must consider the unusual characteristics of EUS images. Compared with normal images, many EUS images, at least reported in this work, contain a significant level of “speckle-noise”, an inherent disadvantage of EUS by the scattering of ultrasound waves within tissues/medians. The speckle noise manifests as random bright and dark textures with low contrast and irregular distribution. Such speckle noise introduces confusion into the convolutional algorithms with meaningless features or boundaries and significantly increases the probability that the model is susceptible to overfitting.

Therefore, good models must possess the capability to exclude features from speckle noise which comprises the majority of true features in many EUS images. Based on our experiment in model tuning, there are three critical ingredients to suppress the model from picking up noise as features: using a large dropout rate, incorporating batch normalization, and applying L2 regularization (with the weight decay parameter set to 0.00001).

**Figure 6:**
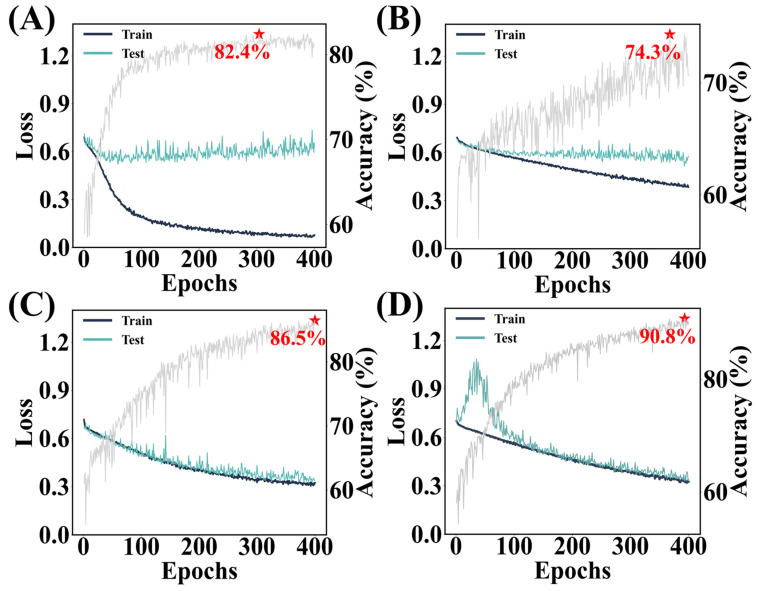
(**A**) Impact of adjusting the dropout rate on model performance. (**B**) Learning curve of the model without batch normalization. The learning curve of the model (**C**) without L2 regularization vs. (**D**) with L2 regularization over 400 training epochs. Grey lines represent the accuracies evolves along with the epoch numbers. Asterisks represent the accuracy values that the models can reach on the validation set.

First, using a large dropout rate forces the true features to compete with the noise features and only true features significantly contribute to reducing the cost function. In Figure 6A, a model with a *p* = 0.5 dropout rate (a common rate seen in many models) shows an overfitting pattern which is due to picking up noise features. Therefore, with a limited number of EUS images, at least a *p* = 0.8 dropout rate is needed to encourage the model to ignore the noise features and pursue a lower cost.

Second, batch normalization is critical in aligning features among images. As true features extracted by convolutional layers, the distribution of features varies from batch to batch due to the complicated nature of GISTs/leiomyomas as significant internal covariate shifts. Using batch normalization layers is complementary. Figure 6B shows the impact of removing batch normalization while retaining a *p* = 0.8 dropout rate and weight decay.

Last, the weight decay serves as the regularization term allowing the model to focus more on the true features and ignore noise features. Figure 6C demonstrates the effect of removing weight decay, which resulted in slightly lower test accuracy compared to Figure 6D, indicating weight decay reduces reliance on irrelevant features and enhances generalization.

As in the lightweight model, the physics-oriented noise can be sufficiently considered and handled with proper machine learning techniques. The architecture can be better constructed to match the needs in reality.

### 4.3. Benchmarks and Outlook: A Deep Model or a Lightweight Model?

In this work, a lightweight model based on EUS images for the classification of GISTs and leiomyomas is reported. Unlike previous models that relied on convolutional layers for feature extraction and classification [22], the present model is distinguished by its noise feature control for limited datasets, achieving ideal performance. Furthermore, the model exhibits consistent performance across different lesion sizes, outperforming prior models [23]. Table 4 summarizes the key parameters and performance metrics of various studies, including the current one. It is more common to see large models than small models based on results from less than 100 to several hundred patients.

**Figure 7:**
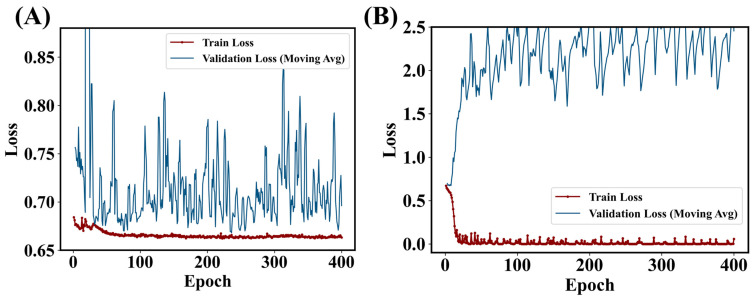
Training and validation loss curves for 2-class ResNet-18 with the weight decay parameter set to (**A**) 0.05 and (**B**) 0.00001. The overfitting is too significant to be suppressed by regularization.

By visually comparing EUS and social media images, variances among EUS images should be much smaller than social media images. For instance, as common characteristics in EUS images, there is always a central cylinder with encircled tissues/liquids. In contrast, it is hard to imagine a common pattern exists across social media images. Therefore, compared with large models which are designated for general images, the lightweight model may be just right for the EUS task. To support our perspective of the comparison between a large model and the lightweight model, a ResNet-18 model is adopted and tuned with only the last classifier modified to a 2-class classifier. Each fine-tuning of the ResNet-18 model is usually accompanied by a significant overfitting trend. To suppress the overfitting trend with hyperparameters, the weight decay parameter (regularization) must be set to a large value of 0.05 (compared to 0.0001 in the lightweight model). In the scheduler, the initial learning rate is set to 0.0005 and is halved every 20 epochs, with training conducted for a total of 400 epochs. As demonstrated in the loss curve (Figure 7A), when the training loss decreased minimally, the validation loss exhibited severe oscillations. A large regularization and unstable test loss clearly show the overfitting issues in the ResNet-18 model. If the weight decay is small enough (Figure 7B) the training and validation loss diverge.

To understand the model complexity, the number of model parameters is counted. The lightweight model contains only 1,434,658 parameters, compared to the 11,171,266 parameters of 2-class ResNet-18. Thus, the best model reported in this work is a “lightweight” model for its significantly fewer model parameters. Considering the smaller variance across EUS images (than common images), there may be no need to use 11 million parameters. When one focuses on the ResNet-50 or EfficientNet architecture reported [25,27], it is difficult to tell if the noise in EUS images is trimmed and not captured by model parameters. Besides, if we limit our EUS dataset to a regional one, the lightweight models may be the practical solution to EUS reading. We know that each machine learning model (even the linear regression model) can be interpreted as a hypothesis with learnable parameters. So, according to Occam’s Razor, when multiple hypotheses can equally explain a phenomenon, the simplest one should be chosen, i.e., “entities should not be multiplied beyond necessity”. Particularly for EUS reading, when a lightweight model and a deeper model both work, the one with fewer model parameters should be the best bet.

## 5. Conclusions

This study proposes a lightweight CNN model optimized for the classification of GISTs and leiomyomas using EUS images. The dataset comprises 13,277 augmented grayscale images from 703 patients and is used to train and evaluate the model through five-fold cross-validation. The optimized model architecture consists of seven convolutional layers followed by fully connected layers. On an independent test set, the model achieves an accuracy of 94.5%, with sensitivity, specificity, PPV, and NPV of 93.9%, 95.4%, 96.9%, and 91.1%, respectively. Compared to human endoscopists, the model demonstrates superior diagnostic performance, particularly in challenging cases where lesion size is ≥20 mm. The study highlights the advantages of lightweight models in EUS image analysis, showing that a model with fewer parameters effectively suppresses speckle noise while maintaining high classification accuracy. Visualization via Grad-CAM reveals that the model extracts more comprehensive lesion features than human experts, enhancing its diagnostic reliability. The results underscore the potential of lightweight deep learning models as a practical and efficient tool for AI-assisted GIST diagnosis in clinical settings.

## Figures and Tables

**Table 1:** Baseline characteristics of patients (2014–2020 and 2022–2024).

	Training and Validation Dataset (2014–2020)		Test Dataset (2022–2024)
Basic information	Age (years, mean ± SD)	56.26 ± 9.89	57.59 ± 10.74
	Male	249 (36.6%)	10 (45.5%)
	Female	432 (63.4%)	12 (54.5%)
Lesion size	Total (mean ± SD)	9.3 ± 5.7 mm	18.7 ± 10.7 mm
	size < 20 mm	636 (93.4%)	14 (63.64%)
	size ≥ 20 mm	45 (6.6%)	8 (36.36%)
Location	Esophagus	226 (33.2%)	4 (18.2%)
	Cardia	33 (4.8%)	1 (4.5%)
	Fundus	337 (49.5%)	11 (50%)
	Body	85 (12.5%)	6 (27.3%)
Gastric wall layers	Mucosa	91 (13.4%)	2 (9.1%)
	Submucosa	18 (2.6%)	1 (4.5%)
	Muscularis propria	572 (84%)	19 (86.4%)

**Table 2:** Layer-wise Parameters of the Lightweight Model.

Layer Name	Kernel Size (Stride, Padding)	Channels	Output Size	Pooling Layers	Parameters
Conv1	9 × 9 (1,0)	1→32	352 × 352 × 32	AvgPool (2 × 2,2)	2624
Conv2	5 × 5 (1,0)	32→32	172 × 172 × 32	AvgPool (2 × 2,2)	25,632
Conv3	5 × 5 (1,0)	32→64	82 × 82 × 64	AvgPool (2 × 2,2)	51,264
Conv4	5 × 5 (1,0)	64→128	37 × 37 × 128	AvgPool (2 × 2,2)	204,928
Conv5	3 × 3 (1,0)	128→256	16 × 16 × 256	AvgPool (2 × 2,2)	295,168
Conv6	3 × 3 (1,0)	256→256	6 × 6 × 256	AvgPool (2 × 2,2)	590,080
Conv7	2 × 2 (1,0)	256→256	2 × 2 × 256	AvgPool (2 × 2,2)	262,400
FC1	-	256→2	2	-	514

**Table 3:** Comparison of performance metrics between the best lightweight model and endoscopists for distinguishing GISTs from leiomyomas on the internal test set (per image).

	Sensitivity %	Specificity %	Accuracy %	PPV %	NPV %
Performance (validation set)	97.7	94.7	96.2	94.6	97.7
Performance * (test set)	93.9	95.4	94.5	96.9	91.1 **
Endoscopists	55.6	79.6	67.6	73.2	64.2

* The performance of the lightweight model on the test set is worse than its performance on the training set. Considering the lesion size significance of the test set, we believe the model’s performance shows its potential in the application in clinical services. ** The unbalance of PPV-NPV is magnified by the unbalanced GIST-leiomyoma ratio (66:43) in the test set.

**Table 4:** Model performance comparison.

Approaches	Authors	Goals	Dataset	Accuracy %	References
Random Forest	Joo et al.	GIST vs. Non-GIST	464 patients	89.6	[24]
Xception	Minoda et al.	GIST vs. Non-GIST	273 patients	SELs ≥ 20 mm: 90.0, SELs < 20 mm: 86.3	[16]
ResNet-50	Tanaka et al.	GIST vs. Leiomyoma	53 patients	90.6	[25]
EfficientNetV2-L	Hirai et al.	GIST vs. Non-GIST	664 patients	89.3	[26]
ResNet-50	Yang et al.	GIST vs. Leiomyoma	752 patients	Internal: 96.2, External: 66.0	[27]
EfficientNet	Oh et al.	GIST vs. Leiomyoma	168 patients	92.3	[28]
ResNet-50	Seven et al.	GIST vs. Leiomyoma	145 patients	86.98	[29]
CNN	Kim et al.	GIST vs. Non-GIST	248 patients	79.2	[22]
CNN	This model	GIST vs. Leiomyoma	703 patients	Validation: 96.2 Test: 94.5	This work

## Data Availability

Restrictions apply to the datasets. The datasets presented in this article are not readily available because the data are part of an ongoing study.
